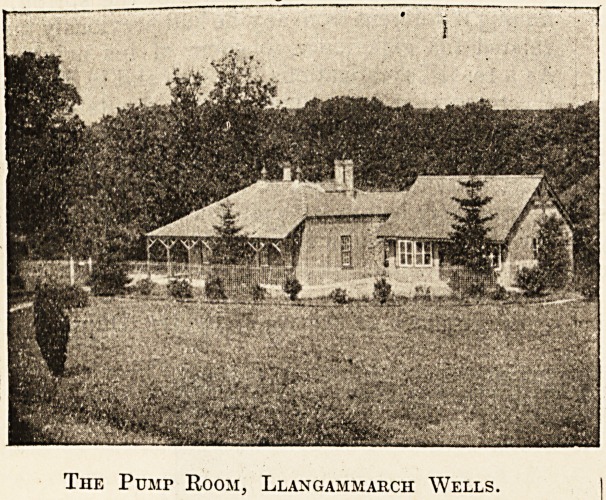# Home and Foreign Spas
*Previous articles of this series appeared in The Hospital of Jan. 28, Feb. 25, March 25, April 22, May 20, June 3, June 17, and July 8.


**Published:** 1911-07-29

**Authors:** 


### SPECIAL ARTICLE.

### HOME AND FOREIGN SPAS.*

### IX.—LLANGAMMARCH WELLS.

The little village or hamlet of Llangammarch Wells, on the banks of the beautiful river Irfon in the County of Brecon, is delightfully situated amid gently sloping well-timbered hills and surrounded by miles of moorland and mountain, affording some of the most charming scenery to be found in South Wales.

The Spa is about 580 feet above sea-level and bounded on the south side by the Eppynt Mountain, rising to an altitude of over 1,500 feet. It is well sheltered and comparatively free from extremes of temperature, the prevailing winds being from south-west. The air, in consequence of the remoteness of the Spa from manufacturing centres, is peculiarly pure and invigorating, and although the average rainfall is fairly heavy, it is by no means humid, but possesses that exhilarating crispness typical of the Alps. The sanitary arrangements are quite modern and on approved principles, and the drinking water is taken direct from one of the mountain streams.

Llangammarch Wells from the Hills.

Llangammarch is on the Central Wales branch of the London and North Western Railway nearly midway between Shrewsbury and Swansea. It is some 213 miles from London and is reached in about five and a half hours; there are several trains daily to and from Euston, to which through carriages are attached so that the journey can be accomplished without inconvenience.

#### The Spring.

The discovery of the mineral water of Llangammarch, like that of many other spas, was accidental. In 1832, when the bed of the Irfon was nearly dry owing to drought, a labourer from a neighbouring farm was hunting for a stray pig which he found in a pool close by a spring which was issuing from the river bank some feet below the usual water-level. The labourer drank of the water of the spring, and finding it different from any he had previously tasted, related his experience to some of his neighbours. As a result investigations followed and in due course the mineral water gained a local reputation in the treatment of various disorders, and more particularly in chronic affections of the heart. It was not, however, until the question of balneo-therapeutics came prominently before the profession in consequence of the excellent results obtained by Schott at Nauheim that the barium spring of Llangammarch claimed serious attention.

An analysis of the water by Dr. Dupré in 1883 and the *Lancet* in 1894 shows it to contain chlorides of sodium, calcium, magnesium, lithium, ammonium, and, above all, barium, to the extent of some 6½ grains to the gallon; there are also distinct traces of bromine. Owing to the fact that chloride of barium is precipitated by sulphates the presence of this salt in mineral water is quite exceptional. It has been found in certain mines in the North of England, but it is associated with such an excessive amount of common salt that it is undrinkable. Consequently the barium spring of Llangammarch is quite unique, anyhow so far as this country is concerned.

#### The Pump Room and Baths.

The Pump Room, which is built immediately over the barium spring, is situated in the beautiful and extensive grounds of the Lake Hotel, nearly a mile

* Previous articles of this series appeared in The Hospital of Jan. 28, Feb. 25, March 25, April 22, May 20, June 3, June 17, and July 8.

from the village. Here also until recently was the bathing establishment. The Baths, which are fitted with the most modern improvements, now occupy an annexe of the hotel, a great convenience to residents without rendering them any the less accessible to non-residents.

The mineral water of the Llangammarch spring has proved of the greatest assistance in the treatment of cardiac diseases, including dilatation and hyperthropy, angina pectoris, and irritable heart following attacks of influenza. It is also beneficial in neurasthenia and various rheumatic and gouty affections, and even cases of Graves' disease show improvement under its influence. The water is administered both internally and in the form of baths, such as reclining bath, douche, shower, needle and wave bath. The methods adopted are similar to those carried out at Nauheim and include massage, resistance exercises, and hill climbing, whilst particular attention is given to diet. Llangammarch has however the advantage of being in our own island and consequently more accessible; moreover, its climate is more bracing than Nauheim, which does away with the necessity for an aftercure.

The Pump Room, Llangammarch Wells.

The Medical Department of the Spa is under the direction of Dr. Black Jones, who has been in residence some fifteen years, during which period he has reported several cases of particular interest to the profession through the medium of the medical press.

### Accommodation and Amusements.

The principal hotel is the Lake, standing in its own ornamental grounds at an altitude of 600 feet on the slope of the Eppynt Mountain and within a hundred yards of the Pump Room. It has lately been considerably enlarged, is lighted throughout with electricity, and is very comfortable and homelike. Then there is the Bungalow Hotel, which is a little less expensive, and several boarding and apartment houses where accommodation can be obtained to suit all purses.

As the season extends from spring to autumn the question of outdoor recreation only need concern us here, although private entertainments are given from time to time at the Lake Hotel. There is an excellent golf course of eighteen holes within a few minutes' walk of the Spa, forming perhaps one of the most interesting and sporting courses to be found south of the Tweed. There are also tennis and croquet lawns, bowling greens, two miles of salmon and trout fishing in the Irfon, and boating and canoeing on the lake. Rough shooting over several hundreds of acres of mountain and moorland is reserved to visitors, and as the roads are good motoring to places of interest in the neighbourhood can be thoroughly enjoyed.

## Figures and Tables

**Figure f1:**
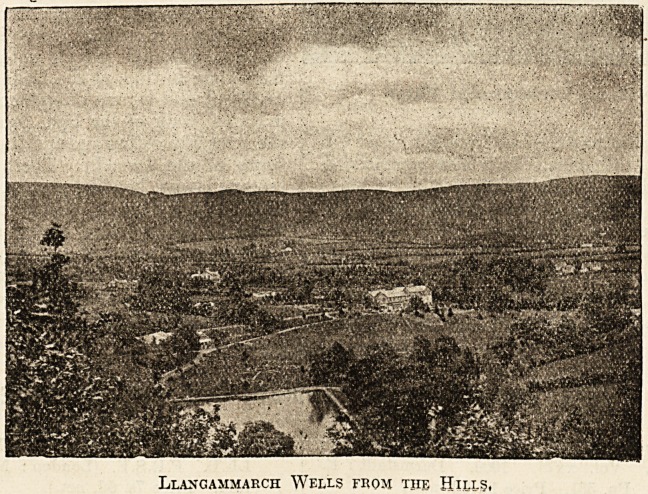


**Figure f2:**